# Reliability and validity of the Chinese version of the biological rhythms interview of assessment in neuropsychiatry in patients with major depressive disorder

**DOI:** 10.1186/s12888-022-04487-w

**Published:** 2022-12-29

**Authors:** Shen He, Lei Ding, Kaibing He, Baoying Zheng, Dan Liu, Min Zhang, Yao Yang, Yingqun Mo, Hua Li, Yiyun Cai, Daihui Peng

**Affiliations:** 1 Division of Mood Disorders, Shanghai Mental Health Center, Shanghai Jiao Tong University School of Medicine, 600 South Wan Ping Road, Shanghai, 200030 People’s Republic of China; 2 Shanghai Medical College of Fudan University, Shanghai, China; 3 Hospital Tuanku Jaafar, Seremban, Malaysia

**Keywords:** Major depressive disorder, Biological rhythm, C-BRIAN

## Abstract

**Background:**

Although disturbances in biological rhythms are closely related to the onset of major depressive disorder (MDD), they are not commonly assessed in Chinese clinical practice. The Biological Rhythms Interview of Assessment in Neuropsychiatry (BRIAN) has been used to evaluate disturbances in biological rhythms in MDD. We aimed to assess and confirm the reliability and validity of the Chinese version of the BRIAN (C-BRIAN) in patients with MDD.

**Methods:**

A total of 120 patients with MDD and 40 age- and sex-matched controls were recruited consecutively. Reliability was estimated using Cronbach’s alpha, the split-half coefficient, and the test-retest coefficient; test-retest reliability was assessed using Spearman’s correlation coefficient. A confirmatory factor analysis was used to determine the construct validity of the scale. The Pittsburgh Sleep Quality Index (PSQI) and the Morningness-Eveningness Questionnaire (MEQ) were used to check concurrent validity by evaluating the correlation between the C-BRIAN, PSQI, and MEQ.

**Results:**

The overall Cronbach’s α value was 0.898, indicating good internal consistency. The Guttman split-half coefficient was 0.792, indicating good split-half reliability. Moreover, the test-retest reliability for both the total and individual item score was excellent. Confirmatory factor analysis revealed that construct validity was acceptable (χ2/df = 2.117, GFI = 0.80, AGFI = 0.87, CFI = 0.848, and RMSEA = 0.097). Furthermore, total BRIAN scores were found to be negatively correlated with MEQ (*r* = **−** 0.517, *P* < 0.001) and positively correlated with PSQI (*r* = 0.586, *P* < 0.001). In addition, patients with MDD had higher BRIAN scores than those in controls.

**Conclusions:**

This study revealed that the C-BRIAN scale has great validity and reliability in evaluating the disturbance of biological rhythms in patients with MDD.

## Background

Biological rhythm, also known as the biological clock, is a behavioral and psychological phenomenon that fluctuates periodically in living organisms and regulates sleep-wake and rest-activity patterns, feeding behavior, energy metabolism, and hormone secretion [1]. Disturbances in biological rhythms impair mood, behavior, and cognition and may lead to mood disorders [2].

Disturbances in biological rhythms have been widely observed in patients with major depressive disorder (MDD) [3] . In addition to low mood, decreased interest, and energy, MDD is often accompanied by disturbances in biological rhythms, such as early morning waking, changes in sleep patterns, daily food intake, and diurnal mood [4]*.* Moreover, disturbance of biological rhythms could lead to the onset and recurrence of MDD, which is an important clinical feature [5] . Clinical studies have demonstrated that suicidal ideation and the severity of depression are associated with the degree of dysregulation of biological rhythms [6, 7]. The most effective treatments for MDD, such as antidepressants (SSRIs, SNRIs, and agomelatine), bright therapy, and social rhythm therapy, can directly affect biological rhythms [8]*.* Basic scientific evidence supports a direct link between disturbed biological rhythms and depressive-like behavior [9, 10] .

There are abundant alterations in circadian gene expression patterns in the postmortem brains of patients with MDD, particularly in the canonical clock genes [11]. Furthermore, the knockout of the circadian gene *Per2* disturbs corticosterone secretion and leads to depressive-like behaviors [12]. Therefore, the study of circadian rhythm is crucial not only for exploring the pathogenesis but also for the treatment of MDD.

Although some tools, such as PSQI and MEQ, have been used to evaluate biological rhythms in clinical practice [13, 14], they mainly focus on assessing sleep disturbances. They cannot provide a comprehensive evaluation of biological rhythms, such as social rhythms. The Biological Rhythms Interview of Assessment in Neuropsychiatry (BRIAN) scale was developed to comprehensively assess biological rhythms, including sleep, social, activity, and eating patterns, in bipolar disorder in a clinical setting [15].

Sleep patterns, daily food intake, activity and social rhythms, as individual endogenous circadian rhythm patterns, have been reported to influence release of hormones (melatonin) and/or some of the major neurotransmitters (Dopamine, noradrenaline and serotonin) implicated in mood regulation in MDD [16–19]. For example, dopamine, noradrenaline and serotonin are known to be modulated by exercise [20]. Thus, it is reasonable to expect the correlation between disturbance of circadian rhythm displayed by BRIAN scale and pathological mechanisms underlying in MDD.

Many previous studies have used it to assess biological rhythm dysfunction in patients with bipolar disorder or MDD [21–27]. However, to the best of our knowledge, no previous study has investigated the psychometric properties of the BRIAN scale for MDD. Thus, this study aimed to assess the reliability and validity of this method in Chinese patients with MDD.

## Methods

### Participants

A total of 120 patients with MDD, as defined by a clinical diagnosis made by a specialized attending physician according to the Diagnostic and Statistical Manual of Mental Disorders, Fifth Edition (DSM-5), were recruited for this study at the Shanghai Mental Health Center between December 2021 and September 2022. Also, 40 age- and sex-matched healthy controls were consecutively recruited from the general population of the Shanghai Mental Health Center. Healthy controls had no family history of MDD or other mental disorders in first-degree relatives. The study was approved by institutional review board of the Shanghai Mental Health Center and conducted in accordance with the Declaration of Helsinki. All participants agreed to participate in the study and provided written informed consent.

### Assessments

The BRIAN is an assessment tool designed to be administered by clinicians. It comprises 18 items that can be classified into the following four main domains related to biological rhythm disturbances: sleep, activity, social rhythms, and eating patterns. All items were evaluated on a 4-point scale (ranging from 1 to 4 points). The total BRIAN score ranged from 18 to 72. A higher score suggested a more severe biological rhythm disturbance. The BRIAN scale has been validated in many different languages, including English, Portuguese, Spanish, Italian, and Korean, to assess biological rhythm in patients with bipolar disorder [28, 29].

The BRIAN scale was translated from English into Chinese using the standard forward-backward translation procedure. We obtained approval from the original author, Dr. Kapczinski, before translation. Dr. Kapczinski and Dr. Adriane provided expert reviews throughout the translation process. Patients with MDD were simultaneously evaluated using the MEQ and PSQI. These scales were used to compare the abilities to determine chronotypes and concurrent validity, similar to previous studies [15, 29]. A subgroup of 30 patients completed a retest and was evaluated twice at 1-week intervals, allowing the exploration of retest reliability.

### Statistical analyses

Reliability was evaluated in three aspects (internal consistency, split-half reliability, and test-retest reliability). Internal consistency was assessed using Cronbach’s α coefficient. The test-retest reliability was analyzed using Spearman’s correlation. Split-half reliability was determined using the Guttman coefficient. We also evaluated the construct and criterion validity of the Chinese version of the BRIAN (C-BRIAN). The MEQ and PSQI were used to test the criterion validity. Confirmatory factor analysis was performed to determine construct validity using the Statistical Product and Service Software Automatically tool (SPSSAU) (retrieved from https://www.spssau.com), which has also been used in previous studies [30–32]. All other statistical analyses were conducted using SPSS version 23.0 (IBM Corp., Armonk, NY, USA). Shapiro-Wilk test was used to check if a continuous variable follows a normal distribution. We found that age, MEQ total score, PSQI total score and CBRIAN total score and the four domain scores were not normally distributed in either one group or both groups. Thus, the Mann–Whitney U test was conducted to compare the data of the two groups. The receiver operating characteristic (ROC) curve was used to determine the cutoff point of the test for identifying MDD. Statistical significance was set at *p* < 0.05.

## Results

### Participant characteristics

A total of 120 patients with MDD and 40 healthy controls were recruited for this study (Table 1). The age of patients with MDD and controls was 31.10 ± 17.25 and 29.67 ± 6.81 years, respectively. There were no significant differences in age and sex (*p* = 0.068 and *p* = 0.853, respectively) between both groups.

**Table 1:** Characteristics of participants

	MDD(*n* = 120)	Healthy controls(*n* = 40)	*P* value
age	31.10 ± 17.25	29.67 ± 6.81	0.068
Sex (male/female)	49/71	17/23	0.853
C-BRIAN total score	39.53 ± 12.02	26.43 ± 6.97	<0.001
MEQ total score	46.83 ± 11.88	49.07 ± 12.17	0.222
PSQI total score	10.13 ± 4.50	0.85 ± 0.80	<0.001

### Internal consistency and test-retest reliability

Cronbach’s α for the C-BRIAN was 0.898. Cronbach’s α values for each item are listed in Table 2. Furthermore, the Guttman split-half coefficient was 0.792. The data showed good internal consistency. To determine test-retest reliability, a subgroup of 30 patients completed a retest of the C-BRIAN within 1 week of the first assessment. The test-retest correlation coefficient for the total BRIAN score was 0.887 (*p* < 0.001).

**Table 2:** Internal Consistency of C-BRIAN Items

BRIAN items	Scale mean if item deleted	Corrected item total correlation	Cronbach’s alpha if item deleted
BRIAN item 1	36.8333	.0.370	.898
BRIAN item 2	36.9833	.603	.891
BRIAN item 3	37.2250	.549	.892
BRIAN item 4	36.9833	.591	.891
BRIAN item 5	36.9917	.504	.894
BRIAN item 6	37.0083	.584	.891
BRIAN item 7	37.2833	.614	.890
BRIAN item 8	37.2000	.635	.890
BRIAN item 9	37.2250	.693	.888
BRIAN item 10	37.7083	.428	.896
BRIAN item 11	37.4500	.639	.890
BRIAN item 12	37.8583	.225	.901
BRIAN item 13	37.2167	.653	.889
BRIAN item 14	37.4083	.535	.893
BRIAN item 15	37.5583	.578	.892
BRIAN item 16	37.3417	.634	.890
BRIAN item 17	37.3917	.548	.892
BRIAN item 18	37.9750	.309	.899

### Structural validity

Confirmatory factor analyses were performed for the four-factor model of the C-BRIAN to access the fit indices. The acceptability criteria of the model fitness indicators are as follows: AGFI, GFI, and CFI equal to or > 0.8. RMSEA between 0.08 and 0.1 indicated acceptability [33, 34]. The model fit showed acceptable fit indices (χ2/df = 2.117, GFI = 0.80, AGFI = 0.87, CFI = 0.848, and RMSEA = 0.097).

### Construct validity

Spearman correlation analysis showed a significant correlation between total BRIAN and MEQ and PSQI scores (*r* = − 0.517, *p* < 0.001; *r* = 0.586, *p* < 0.001), respectively. The results showed a statistically significant, moderate association.

### Comparison of C-BRIAN scores between patients with MDD and healthy controls and cutoff values of C-BRIAN

**Fig. 1:**
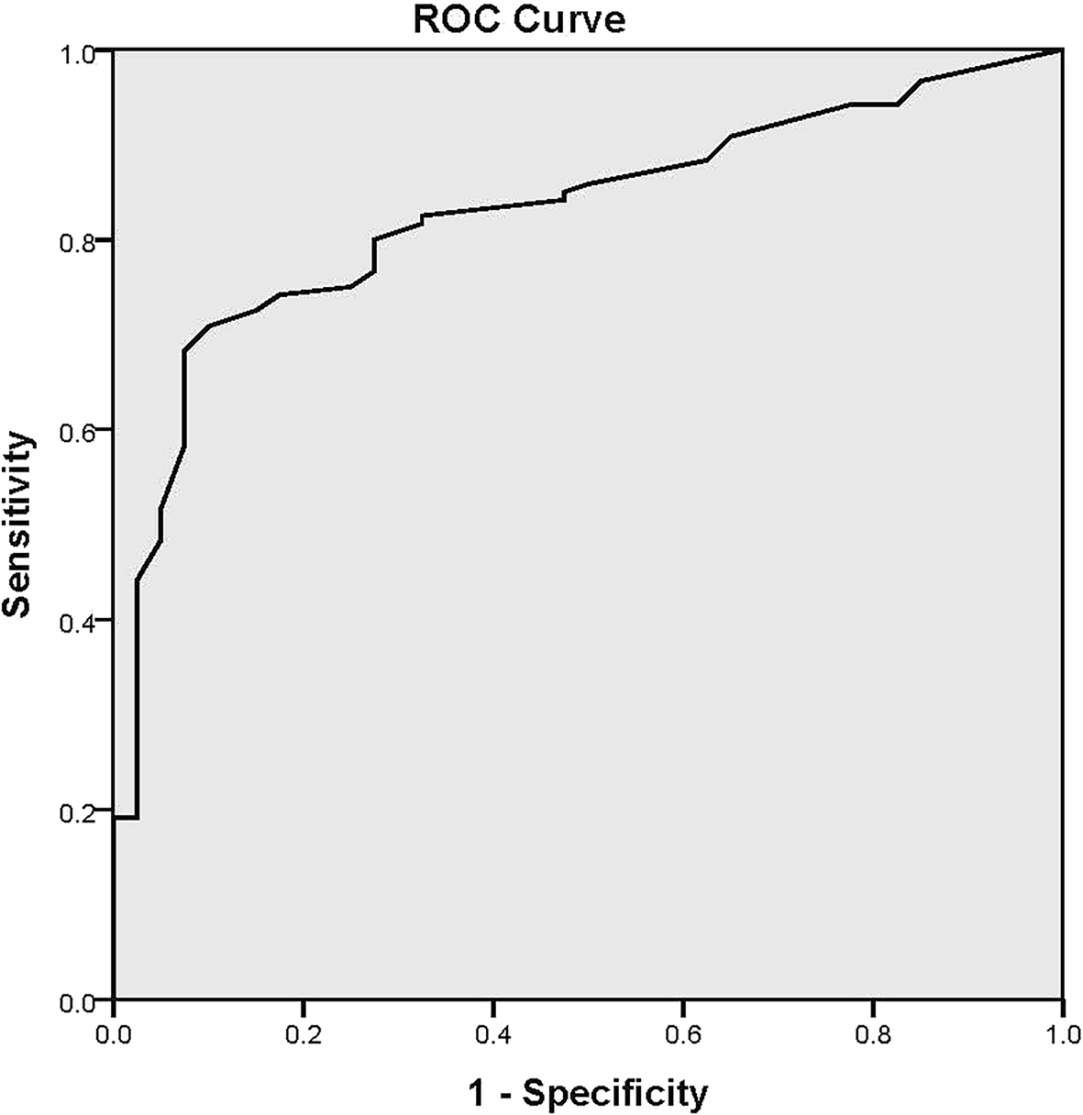
Results of the area under the receiver operating characteristic curve analysis for the C-BRIAN

Total BRIAN scores of patients with MDD and healthy controls were 39.53 ± 12.02 and 26.43 ± 6.97, respectively (Table 1). The Mann–Whitney U test showed that the total C-BRIAN scores in patients with MDD were significantly higher than those in healthy controls (Z = -6.221, *p* < 0.001). Higher BRIAN scores indicate a more disrupted circadian rhythm. Moreover, all four domains of the BRIAN scores were higher in patients with MDD than they were in healthy controls (all *p* < 0.001) (Table 3). Furthermore, ROC analysis showed a good performance in screening between patients with MDD and healthy controls (AUC = 0.829, 95% CI =0.762–0.895) for BRIAN (Fig. 1). The cutoff point for BRIAN was ≥35, with the best combination of sensitivity and specificity (70.8 and 90.0%, respectively).

**Table 3:** Mean scores on the domains of the C-BRIAN

Domains	MDD	controls
Sleep	12.53 ± 4.08	8.23 ± 3.09
Activity	11.12 ± 4.28	7.00 ± 1.85
Social rhythms	8.13 ± 3.10	5.70 ± 1.70
Eating pattern	7.77 ± 3.34	5.50 ± 2.04

## Discussion

The BRIAN scale is a simple tool for comprehensively assessing disturbances in biological rhythms. Patients with MDD often have altered biological rhythms, including changes in the sleep-wake cycle, exercise, diet, and social interactions [4]. However, the previously commonly used scales, such as the MEQ and PSQI, can only be evaluated in the sleep dimension; therefore, a scale that can extensively evaluate biological rhythm is also required for scientific research or clinical practice in patients with MDD. The BRIAN scale has been used to assess biological rhythms in patients with MDD [25–27]. Using this scale, researchers have observed that biological rhythm disruption is associated with the severity of depression [6, 35]. However, to date, no research has examined the reliability and validity of the BRIAN scale in patients with MDD. To the best of our knowledge, this is the first study to translate the BRIAN scale into the Chinese language and show that the C-BRIAN is a valid and reliable measure with good reliability and validity for patients with MDD.

Overall, the results of this study demonstrate that the C-BRIAN scale has good internal consistency, convergent, and test-rerest reliability, which is similar to the results of previous BRIAN studies. The results also showed that the internal consistency of the C-BRIAN was good and similar to those of the Korean and Italian versions [23, 24]. Similar to previous studies, there were significant correlations between the total BRIAN, MEQ, and PSQI scores, indicating good convergent validity [15, 28].

Furthermore, we found higher BRIAN scores in patients with MDD than were found in healthy controls. Our results indicated that the C-BRIAN scale showed good discriminant validity in screening for MDD. The area under the ROC curve was > 0.8. This is a relatively high value, indicating excellent diagnostic ability for MDD. In this study, we had a putative cutoff point ≥ 35 because this value showed the best discriminant effects for diagnosing MDD with a sensitivity of 70.8% and a specificity of 90%. This may provide a new screening tool for detecting MDD. It should be emphasized that BRIAN was not originally designed or tested as a screening tool; however, it showed acceptable screening features.

This study had some limitations. First, although the study sample was larger than some previous studies on the reliability and validity of the BRIAN scale, the sample size was still relatively small. Second, all participants in this study were recruited from only one hospital in Shanghai, which may have led to a selection bias. Therefore, this sample may not represent all populations in China. In future studies, the sample size should be increased and patients from other regions of China should be included to increase representativeness. Moreover, it is necessary to further examine the correlation between rhythm disturbance and severity of depression, anxiety, suicide risk and melatonin level in Chinese patients with MDD.

## Conclusions

We confirmed the validity and reliability of the C-BRIAN in the Chinese population. Moreover, a significantly higher C-BRIAN score was found in patients with MDD than the scores in the healthy controls. In the future, we hope to promote the clinical application of this scale to patients with MDD in the Chinese population nationwide. This will facilitate a more comprehensive assessment of the clinical symptoms of patients with MDD in China.

## Data Availability

The datasets used and/or analyzed during the current study are available from the corresponding author on reasonable request.
